# Visual Attention Measures Predict Pedestrian Detection in Central Field Loss: A Pilot Study

**DOI:** 10.1371/journal.pone.0089381

**Published:** 2014-02-18

**Authors:** Concetta F. Alberti, Todd Horowitz, P. Matthew Bronstad, Alex R. Bowers

**Affiliations:** 1 Schepens Eye Research Institute, Massachusetts Eye and Ear, Department of Ophthalmology, Harvard Medical School, Boston, Massachusetts, United States of America; 2 Basic Biobehavioral and Psychological Sciences Branch, Behavioral Research Program Division of Cancer Control and Population Sciences, National Cancer Institute, Rockville, Maryland, United States of America; University of British Columbia, Canada

## Abstract

**Purpose:**

The ability of visually impaired people to deploy attention effectively to maximize use of their residual vision in dynamic situations is fundamental to safe mobility. We conducted a pilot study to evaluate whether tests of dynamic attention (multiple object tracking; MOT) and static attention (Useful Field of View; UFOV) were predictive of the ability of people with central field loss (CFL) to detect pedestrian hazards in simulated driving.

**Methods:**

11 people with bilateral CFL (visual acuity 20/30-20/200) and 11 age-similar normally-sighted drivers participated. Dynamic and static attention were evaluated with brief, computer-based MOT and UFOV tasks, respectively. Dependent variables were the log speed threshold for 60% correct identification of targets (MOT) and the increase in the presentation duration for 75% correct identification of a central target when a concurrent peripheral task was added (UFOV divided and selective attention subtests). Participants drove in a simulator and pressed the horn whenever they detected pedestrians that walked or ran toward the road. The dependent variable was the proportion of timely reactions (could have stopped in time to avoid a collision).

**Results:**

UFOV and MOT performance of CFL participants was poorer than that of controls, and the proportion of timely reactions was also lower (worse) (84% and 97%, respectively; p = 0.001). For CFL participants, higher proportions of timely reactions correlated significantly with higher (better) MOT speed thresholds (r = 0.73, p = 0.01), with better performance on the UFOV divided and selective attention subtests (r = −0.66 and −0.62, respectively, p<0.04), with better contrast sensitivity scores (r = 0.54, p = 0.08) and smaller scotomas (r = −0.60, p = 0.05).

**Conclusions:**

Our results suggest that brief laboratory-based tests of visual attention may provide useful measures of functional visual ability of individuals with CFL relevant to more complex mobility tasks.

## Introduction

Central field loss (CFL) is the presence of scotomas (blind areas) within the central visual field including the fovea. The most common cause of CFL is age-related macular degeneration, a major public health concern with the increasingly aged population. Despite advances in treatments [1], the majority of individuals with macular degeneration have irreversible vision loss that causes difficulties in a range of activities including reading and mobility (walking and driving) [2], [3].

People with CFL almost always use a preferred retinal locus (PRL), an extra-foveal location near the scotoma, to fixate targets that would normally be foveally fixated [4], [5]. Thus they not only have a blind area in central vision, but also impaired visual acuity and contrast sensitivity [6]. In order to compensate effectively for the CFL, the PRL has to be used consistently for fixation, saccades need to be directed to the PRL rather than the fovea, and scanning of a scene with the PRL has to be accomplished in such a way as to minimize occlusion of objects by the scotoma. Results of a prior study in the dynamic environment of a driving simulator suggest that drivers with CFL might not be able to fully compensate for their scotomata, as responses to pedestrian hazards that appeared in scotoma areas were much slower than responses to hazards in non-scotoma areas [7].

In rehabilitation clinics, residual vision of people with CFL is typically evaluated in terms of visual acuity, visual field measures, and, possibly, fixation characteristics and letter contrast sensitivity [6], [8]. However, with the exception of reading, the ability of patients to use their remaining vision for tasks representative of activities of everyday living is rarely evaluated [8], [9]. For example, walking or driving assessments can be time consuming and often require specialized equipment, such as a driving simulator, which might not be available in a clinic. Moreover, traditional vision measures, such as visual acuity and visual fields, typically account for only a small amount of variance in mobility performance of people with CFL [10], [11]. It is therefore important to develop measures of visual performance that are both suitable for implementation in clinical settings and predictive of how well vision is used in real-world tasks (i.e., how well the person compensates for his vision loss).

The ability to deploy visual attention effectively is a fundamental aspect of many tasks, including walking and driving. The Useful Field of View (UFOV) test, the most well-known test of visual attention, has been shown to be predictive of walking and driving performance in elderly populations [12], [13], [14], in visually impaired populations with a broad range of visual deficits [15], [16], and, more specifically, in patients with peripheral field loss [17], [18]. However, only a limited number of studies have included patients with CFL, and they were always part of a larger sample of heterogeneous vision impairments [15], [16].

Driving and walking are carried out in dynamic environments in which both the observer and the objects in the environment are in motion. Attention has to be shifted between objects, divided between objects (attending to the car ahead while being aware of a person about to step into the road) and also maintained for sustained periods of time. For people with CFL, this will require effective gaze strategies and consistent use of the PRL. However, commonly-used tests of visual attention, including the UFOV, typically use only brief presentations of static stimuli. A task requiring sustained attention to moving stimuli, which mimics the continuous attention shifts between objects that are necessary during mobility, might provide a more relevant assessment of visual attentional abilities of patients with CFL than a test of static visual attention. One such task is multiple object tracking (MOT), which measures the ability to track several targets amongst distractors while all are moving in random directions on a computer screen [19].

We conducted a pilot study to examine the effects of CFL on static and dynamic attention, assessed with the UFOV test and a brief MOT test, respectively. We also quantified the ability of each test to predict performance on a more complex mobility-related task in which effective deployment of attention is likely to be important - detection of walking/running pedestrian hazards while driving in a simulator. Participants with CFL who had better scores on the attention tests had better performance on the driving simulator task with a higher proportion of timely responses to the approaching pedestrians than participants who scored less well on the attention tests.

## Methods

### Ethics statement

The study adhered to the tenets of the Declaration of Helsinki and was approved by institutional review boards at the Schepens Eye Research Institute and the Veterans Administration Boston Healthcare System. All participants gave voluntary, written informed consent.

### Participants

Eleven normally-sighted current drivers and eleven people with bilateral CFL participated in the study. They were recruited from Schepens, the Veterans Administration Boston Healthcare System, and the Harvard Cooperative Program on Aging. The normally-sighted group was selected to have a similar age and sex distribution to the CFL group (Table 1). Inclusion criteria for the participants with CFL were: an absolute bilateral central scotoma (involving the fovea) to a 0.7° target at 1 m (kinetic perimetry using a custom computerized central visual fields test [20]), corrected binocular single letter visual acuity of 20/200 or better (the minimum acuity for a restricted driving license in the USA [21]) and at least 120° horizontal binocular field extent (Goldmann perimeter, V4e target). Causes of the CFL included: age-related macular degeneration (n = 6), Stargardt's macular dystrophy (n = 2), optic atrophy (n = 2) and presumed ocular histoplasmosis syndrome (n = 1). Seven of the CFL subjects were current drivers, while four (aged 47 to 81 years) had stopped driving a median of 4 years (range 6 months to 12 years) before being tested in the driving simulator.

**Table 1:** Demographic and visual characteristics of normally-sighted (n = 11) and CFL (n = 11) study participants.

	Normally sighted	CFL	Test for group differences, p-value*
Male; n (%)	8 (72)	6 (54)	0.33
Age, years; Mean (range)	65 (46 to 84)	65 (46 to 87)	0.97
Visual acuity, LogMAR; Mean (range)	−0.05 (−0.12 to 0.12)	0.55 (0.20 to 0.98)	0.001
Contrast sensitivity, log units; Mean (range)	1.81 (1.55 to 1.95)	1.27 (0.90 to 1.60)	0.001
Average binocular scotoma diameter, degrees; Mean (range)	n.a.	12 (5 to 23)	n.a.

^*^Fisher's Exact Test for sex; Student's independent t-tests for other variables.

All participants completed a questionnaire addressing general health and medications to ensure that they did not have any (non-visual) conditions that might affect their simulator performance. Furthermore, none had cognitive decline (all scored at least 9/10 on the Short Portable Mental Status Questionnaire [22]) and none had prior experience of driving in a virtual environment. Habitual spectacle corrections were used for all tests and when driving in the simulator.

### Vision measures

Binocular single letter visual acuity was measured using Test Chart 2000 Pro software (Thomson Software Solutions; Hatfield, Hertfordshire, UK). Binocular letter contrast sensitivity (2.5° letters) was measured with a custom, computer-based test on a luminance-calibrated display [23] that gives results very similar to those obtained with Pelli-Robson and Mars tests for visually impaired patients tested with letters of comparable visual angle (R. Woods, personal communication). The size of the binocular scotoma for each CFL patient was determined under binocular viewing conditions with kinetic perimetry using a custom computerized central visual fields test [20] (0.7° target, similar to the Goldmann IV3d target). The size was calculated as the mean diameter of 4 main meridians passing through the center of the scotoma.

### Visual Attention Tests

The two attention tests were presented on a 20-in touch-screen monitor and were administered at the same session. The UFOV test took 10 to 15 minutes and the MOT test about 20 minutes.

#### Useful Field of View

The three UFOV subtests were administered using the commercially-available UFOV test [24], [25], version 6.0.9 (Visual Awareness Research Group, Inc., Punta Gorda, FL). The first subtest (processing speed) required the identification of a central target (outline of a car or a truck). The second (divided attention) required identification of the central target, as well as localization of a peripheral target (car) presented simultaneously at one of eight radial locations 11 cm from the center of the screen. In the third subtest (selective attention), the central and peripheral targets were embedded among visual distractors (triangles). Targets were displayed from 17 to 500 ms using a double staircase method, and the score for each subtest was expressed as the display duration for which the subject achieved a 75% correct response rate (with longer durations representing poorer performance).

**Figure 1:**
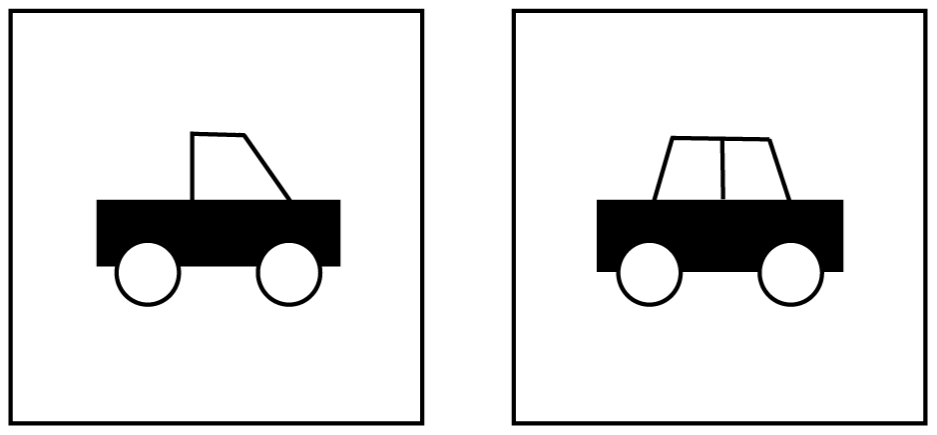
The car and truck targets from the UFOV central identification task. The targets differed only in whether two thin lines were present in the top left area of each outline, making this task difficult for participants with CFL.

In our pilot testing, it quickly became apparent that the central identification task was difficult for the CFL participants as the only difference between the car and truck was a thin line of less than 0.1° width at the recommended 45 cm viewing distance (Figure 1). Prior studies that measured UFOV in patients with CFL used customized software with the ability to alter target size [15], [16]. However, when we conducted this study, only the commercial version of the UFOV test was available for our use, so we could not manipulate the line width and/or size of the central target; the only way to increase target size was by reducing viewing distance. Therefore CFL participants used a preferred distance (median 34 cm, interquartile range 29 to 47 cm) at which they could resolve the detail of the central target. Despite using a shorter viewing distance, the size of the task detail was still closer to the resolution threshold of the CFL participants than of the control participants (the mean visual acuity of CFL participants was 4 times poorer than that of controls (Table 1) but the median decrease in viewing distance only increased the angular thickness of the line by about 1.3 times). Thus, the display durations for CFL participants may have been confounded by differences in central task resolution difficulty, adding variability that was not due to attentional difficulties *per se*. By comparison, the peripheral localization task was much less likely to be affected by visibility issues as both controls and CFL participants performed this task using peripheral vision. Only detection (not identification) of the peripheral target was required and the overall target subtended about 1.9° by 1.3° at 45 cm.

To remove the potentially confounding effects of differences in central task resolution difficulty, the minimum display duration for UFOV subtest 1 was subtracted from those of subtests 2 and 3. These difference scores provided a measure of the effects of the increased demands of the divided and selective peripheral attention tasks, corrected for differences in visibility of the central task. Both the UFOV threshold display durations and the difference scores were analyzed.

One further consideration for the CFL participants was whether any of the peripheral targets might have been obscured by the central scotoma, which would have confounded performance on subtests 2 and 3. Based on their scotoma size and location (as measured with kinetic perimetry described under vision measures), the peripheral targets fell outside of the central scotoma for nine participants. For each of the two remaining participants the peripheral target would have fallen within the scotoma for 2 of the 8 potential locations. These participants were not outliers on any of the UFOV measures.

### Multiple Object Tracking

A brief MOT task designed for clinical populations was administered to all participants [26]. Stimuli were six high-contrast black disks (2 cm in diameter) presented against a lighter grey background. At the start of each trial three target disks were highlighted in green for 2 s, and then turned black again. The task was to track those target disks (among the distractor disks) as they moved for 5–8 s. Each disk had an initial random direction and changed direction when it met the boundaries of the display (23 by 23 cm) or came close to another disk. Thus, disks never occluded one another. At the end of the trial all disks stopped moving and the participant used the touch screen to indicate which disks were the original green targets. Once three disks were selected, feedback was given. The speed at which the disks moved was adjusted on each trial using a simple one-up, one-down staircase. A correct trial was one on which all three targets were selected correctly; a trial with at least one incorrect selection was classified as an incorrect trial. Speed was increased by 40% following correct trials and decreased by 60% following incorrect trials starting from an initial speed of 12°/s. Ten practice trials were followed by 50 test trials and took approximately 20 minutes to complete. For each participant, we then used the QUEST [27] algorithm to estimate the speed (in degrees of visual angle per second) yielding 60% correct performance.

Unlike the UFOV test in which fixation was constrained by the central identification task, CFL and control participants were permitted to move their eyes freely during the MOT task (there was no central fixation target), and to adopt any viewing strategy that might be helpful. Head position was also unrestrained. Participants performed the task at a preferred distance that was comfortable for viewing and interacting with the touch screen. Controls performed the task at a median distance of 52 cm (interquartile range 42 to 53) and CFL participants at a median distance of 40 cm (interquartile range 36 to 43 cm). Thus the 2 cm disks subtended a median of 2.0° for controls and 2.6° for CFL participants. Viewing distance was accounted for in the computation of the threshold tracking speed. Unlike the UFOV central identification task, the MOT task did not require resolution of final detail, only detection of the high contrast disks which were well above the detection threshold for both CFL and control participants. Any differences in disk visibility between CFL and control participants would have had little impact on their ability to track the discs. By comparison, the central scotoma may have impeded performance on the dynamic attention task, but that was what we intended to measure.

### Driving Simulator and Pedestrian Detection task

Each participant completed two driving simulator sessions in a high-fidelity driving simulator (PP-1000, FAAC, Inc., Ann Arbor, MI) with a 225° horizontal field of view and standard controls for a car with automatic transmission [7], [28]. Each session (about one week apart) started with a period of familiarization and practice in the driving simulator. Participants were given as much time as they needed (about 30–45 minutes) to become comfortable controlling the virtual car before progressing to the test drives. Participants then drove three test drives in a city environment (30 mph) and 2 drives on rural undivided highways (60 mph) with other traffic on the road and in the daytime [29], [30]. They had full control of vehicle steering and speed (gas pedal and brake pedal) at all times. Each drive took about 8 to 12 minutes depending on drive length and participant speed. Participants were instructed to follow all the normal rules of the road. Breaks were taken between test drives, as needed.

While driving, participants performed a pedestrian detection task [31]. There were 8–12 pedestrian appearances per drive (52 per session). The initial appearance was at one of four possible eccentricities (−14°, −4°, 4°, 14°) at 67 m (city) and 134 m (highway) from the participant's vehicle. At these distances there was 5 seconds between pedestrian appearance and a potential collision occurring (assuming that the participant was driving at the posted speed limit). Five seconds is twice the perception-brake time (time from hazard detection to first stepping on the brake) used in the calculation of minimum recommended stopping sight distances for safe roadway design [32]. The pedestrians (wearing a grey shirt and trousers) initially vertically subtended 1.5° and 0.75° in city and highway drives, respectively. After appearing, pedestrians walked or ran with biological motion (i.e. their limbs moved realistically) as if to cross the travel lane in front of the approaching vehicle; however, they stopped at the edge of the travel lane to avoid collisions. The speed of the pedestrians was such that there would have been a collision with the participant's car if it had continued without braking and if the pedestrian had continued into the travel lane. Thus pedestrians maintained a relatively constant eccentricity with respect to the car heading direction for most of the approach time. Pedestrians at small eccentricities (−4° and 4°) represented situations in which a pedestrian might approach from an adjacent lane (while crossing the street), or the sidewalk. Pedestrians at larger eccentricities (−14° and 14°) represented hazards approaching more quickly from a greater distance (e.g., a bicyclist). Participants were instructed to press the horn as soon as they saw a pedestrian appear.

### Data analyses

For the attention tests, the following measures were used in analyses: UFOV threshold display durations for subtests 1 to 3, UFOV divided attention difference score (i.e., subtest 2 – subtest 1), UFOV selective attention difference score (subtest 3 – subtest 1) and logarithm of the MOT threshold speed. For ease of interpretation, MOT data are reported in terms of the actual speeds rather than the logarithms of the speeds.

For each pedestrian appearance we calculated whether the participant could have stopped in time to avoid a collision assuming the pedestrian had continued on its trajectory and entered the travel lane. The analysis took account of the participant's speed and distance from the pedestrian at the time when they reacted (pressed the horn). We computed both the time to contact and the time to bring the vehicle to a stop from the time of the horn press. A braking deceleration of 5 m/s^2^ was assumed, representing a dry road and a car in good condition [33]. Each event was then classified as timely or untimely. Timely reactions were those for which participants would have been able to stop in time if they began braking at the time of the horn press (i.e., estimated braking time was less than the calculated time to contact). Untimely reactions were those for which participants would not have been able to stop in time, including events where the braking time was greater than the time to contact, or the pedestrian was not detected. For data pooled across city and highway drives, overall pedestrian detection performance was then summarized as the proportion of all reactions that were timely [31], [34]. To avoid the truncation effect [35], a probit transform was applied to convert the timely reaction proportions to z-scores, which were used in analyses.

Continuous variables were normally distributed and analyzed with parametric tests. The alpha level was 0.05.

## Results

As expected, CFL participants had worse visual acuity and contrast sensitivity scores than participants with normal vision (Table 1). The binocular scotoma diameter ranged from 5 to 23° (Table 1).

The CFL group scored more poorly than controls on all of the attention measures (Table 2). The threshold durations for the UFOV subtests 1, 2 and 3 were significantly higher (longer presentation durations, poorer performance) for the CFL group than controls. The UFOV divided-attention difference scores were also greater for participants with CFL than participants with normal sight indicating a greater impairment in performance for the CFL than the normal vision group in subtest 2 (the divided attention condition) relative to subtest 1 (central discrimination task only). A similar trend was apparent for the selective-attention difference scores, but did not reach significance. In addition, the MOT speed threshold of participants with CFL was lower (worse) than that of participants with normal sight. For the CFL group, UFOV difference scores and MOT speed thresholds were only weakly correlated with vision measures (r<|0.48|, p>0.14) or age (r<|0.36|, p>0.27).

**Table 2:** Mean (SD) performance of normally sighted (n = 11) and CFL (n = 11) participants on each attention test and the simulator detection task.

	Normally sighted	CFL	Test for group differences
**UFOV threshold durations (ms)** ↓			
Subtest 1 central task only	20 (11)	112 (109)	*t*_(20)_ = 2.79, *p* = 0.01
Subtest 2 divided attention	83 (80)	251(113)	*t*_(20)_ = 4, *p* = 0.001
Subtest 3 selective attention	194 (114)	372 (114)	*t*_(20)_ = 3.65, *p* = 0.002
**UFOV difference scores (ms)** ↓			
Divided attention	63 (77)	148 (65)	*t*_(20)_ = 2.79, *p* = 0.01
Selective attention	173 (112)	251(98)	*t*_(20)_ = 1.74, *p* = 0.097
**MOT speed threshold, °/s** ↑	13.5 (4.7)	9.1 (5.2)	*t*_(20)_ = 2.15, *p* = 0.043
**Timely reaction, proportion** ↑	0.96 (.01)	0.84 (.07)	*t*_(19.5)_ = 6.66, *p* = 0.001

↑ Higher scores indicate better performance.

↓ Lower scores indicate better performance.

The proportion of timely reactions in the driving simulator detection task was significantly lower for participants with CFL than participants with normal sight (Table 2). Unlike the UFOV and MOT tasks, in which both groups demonstrated a wide range of performance, in the driving simulator task only the participants with CFL exhibited a range of performance. This was intended; the detection task was designed to be taxing for the visually impaired population but to allow sufficient time for a response to avoid a collision [32].

**Figure 2:**
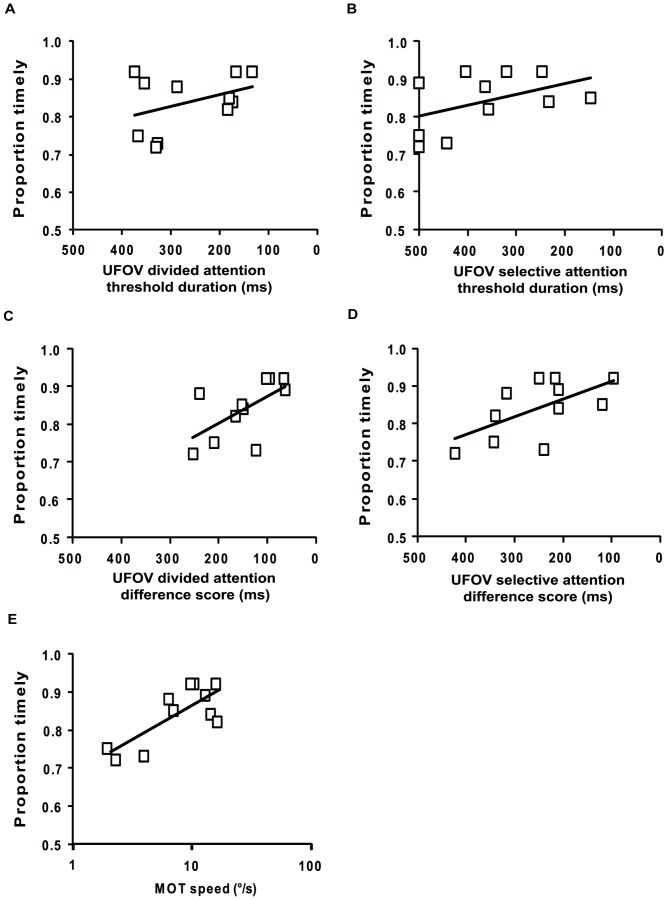
Relationship between proportion of timely reactions and attention measures for CFL participants. **(a)** UFOV divided attention threshold duration, **(b)** UFOV selective attention threshold duration, **(c)** UFOV divided attention difference score, **(d)** UFOV selective attention difference score, and **(e)** the MOT task. Better performance on the pedestrian detection task was associated with better performance on each of the attention tests. UFOV scores are plotted on reversed axes so that better performance is at the right hand side of the x-axis for all figures. Thick black line shows the linear trend.

As participants with normal sight demonstrated such a small range of performance on the simulator detection task, correlations between timely reactions and other measures were only examined for participants with CFL. Higher proportions of timely reactions were significantly correlated with better performance on the attention tests, including: lower difference scores on the UFOV divided and selective attention subtests (r = −0.66, p = 0.03; and r = −0.62, p = 0.04, respectively); and higher MOT speed thresholds (r = 0.73, p = 0.01) (Figure 2). However, correlations with the threshold durations on the UFOV divided and selective attention subtests were not as strong (r = 0.35, p = 0.30; and r = −0.42, p = 0.20, respectively). Higher proportions of timely reactions were also associated with vision measures, including smaller scotomas (r = −0.60, p = 0.05), better contrast sensitivity scores (r = 0.54, p = 0.08) and better visual acuity (r = −0.49, p = 0.15). The proportion of timely reactions was only weakly correlated with age (r = 0.34, p = 0.30) and there was no significant difference in the proportion of timely reactions between current and non-current drivers (t_(9)_ = 1.25, p = 0.24).

We conducted a multiple regression analysis with proportion of timely reactions as the dependent variable (including data from only the CFL participants). In the first step, MOT speed threshold was entered as the only predictor, explaining 48% of the variance (adjusted r^2^ = 0.48, F_(1,9)_ = 10.18, p = 0.01). We then tested whether the model could be improved with the addition of other predictors. As sample size was limited, the model only ever contained MOT speed threshold and one other predictor. Adding UFOV divided attention score accounted for only an additional 1% of the variance (adjusted r^2^ = 0.49, F_(2,8)_ = 5.86, p = 0.03). Similar results were found when each of the other predictors was added (UFOV selective attention difference, visual acuity, contrast sensitivity, and scotoma size); none significantly improved the model that contained only MOT speed threshold.

## Discussion

Participants with CFL demonstrated poorer performance on simulated pedestrian detection, MOT, and greater reductions in UFOV performance in the divided and selective attention conditions (relative to the central-task only condition) than participants with normal sight. This was expected on the basis of prior research on CFL participants in the driving simulator [7]. More interestingly, both MOT and UFOV scores were significant predictors of the ability of CFL participants to detect pedestrians in the driving simulator. CFL participants with a higher proportion of timely reactions to pedestrians could track targets at faster speeds in the MOT task and had less impaired performance in the UFOV divided and selective attention tasks.

For each of the visual attention measures there was a wide range of performance within the CFL group, which was only weakly correlated with vision measures. This suggests that the level of visual impairment *per se* was not an important factor accounting either for variability within the CFL group or for differences between the CFL and normally-sighted groups. Rather it appears that the predominant factors were attentional capacity and the ability to deploy attention in static and dynamic situations (including efficient use of a PRL and compensatory gaze strategies).

Of the visual attention measures, MOT performance had the strongest correlation with performance in the driving simulator detection task. One possibility is that individuals who are better at devising good compensatory strategies (e.g. efficient use of a PRL) might perform well on dynamic tasks such as MOT and pedestrian detection. On this account, the key aspect of the MOT task is its dynamic nature, which taxes the ability to constantly redeploy scarce attentional resources where they are most needed [36]. Similarly, the driving simulator task requires the ability to shift attention where needed, from the speedometer to the road ahead to the side of the road where pedestrians may appear. Note that this ability would be largely useless in static tests of visual attention and static tests of clinical visual function, such as visual acuity or contrast sensitivity [37]. Nevertheless, in agreement with a previous driving simulator study of individuals with CFL [7], timely reactions were also correlated with vision measures, in particular scotoma size and contrast sensitivity, suggesting that clinical assessments of vision for driving should include traditional vision measures as well as measures of the ability to deploy attention. Both MOT and UFOV measure the ability to divide attention. However, there was only a very weak association between performances on the two tasks, which provides support for our hypothesis that MOT adds additional information about attentional abilities in dynamic situations that is not captured by static tests of visual attention.

During the MOT task it is highly likely that some of the targets were obscured by the scotoma at least briefly. Studies of normally-sighted young observers have demonstrated that the visual system can successfully track targets which are briefly occluded [38]. However, tracking through occlusion requires more attentional resources than tracking without occlusion [39]. Thus, tracking may be more attentionally demanding in the presence of a scotoma. More generally, processing resources allocated to compensating for the vision impairment may reduce those available for deployment of attention.

Our study had several limitations. First, we compensated for the reduced visual acuity of our participants by allowing CFL participants to use a preferred working distance, typically less than 45 cm, at which they could resolve the UFOV task detail, rather than adjusting the size of the central target within the software. We also computed alternate attentional performance measures by subtracting the minimum display duration for UFOV subtest 1 (central task only) from those of subtests 2 and 3. While this approach may not be optimal, it was a practical solution which could easily be implemented in vision rehabilitation clinics. Importantly, our results provide evidence in support of this approach to analyzing UFOV data. Specifically, subtraction of the central task-only presentation duration from the other two subtests reduced the variability in the divided and selective attention task scores within the CFL group, but not the control group (Table 2). The reduction was greater for the divided attention than the selective attention task, possibly because the peripheral target was presented against an uncluttered background in the former task but a cluttered background in the latter.

The peripheral target in the UFOV test was at approximately the same retinal eccentricity (about 11°) for all control participants, but varied amongst the CFL group, dependent on the viewing distance and the eccentricity of the retinal location used for fixation. This may have added some additional noise into the UFOV measurements for CFL participants, but was unlikely to have had a major impact on the difficulty of the peripheral localization task as the median decrease in viewing distance was about 1.3 times, which would only have increased the retinal eccentricity to about 14°.

Other limitations of this study include the small sample size and the heterogeneity of the CFL group, which included both current and non-current drivers; however, all were given ample time to practice in the simulator and there were no differences in detection performance between current and non-current drivers. We were not evaluating fitness to drive *per se*; rather, we were using the driving simulator as a safe, controlled, interactive test environment that captured the complexities and attentional demands of a real world mobility task while enabling repeated measurements of detection under the same conditions for all participants; the relationship between detection in simulated and on-road driving has yet to be determined.

Although we cannot draw strong conclusions from this exploratory study, the results are promising. Our findings suggest that our brief laboratory-based test of dynamic visual attention may be a better predictor of simulated driving performance than the UFOV. More generally, dynamic attention tasks may prove to be more useful than static attention tests in measuring the visual performance of individuals with CFL relevant to predicting performance in more complex mobility tasks. A follow-up study is clearly warranted including a larger sample of visually impaired participants, a UFOV test in which the size of the central task detail can be manipulated and tracking of gaze movements to evaluate compensatory scanning abilities. Furthermore, there are many varieties of dynamic attention tests. It is possible that the brief MOT test we utilized might not be the most optimal for evaluation of dynamic sustained attention in individuals with vision impairment; this remains an area for future research.
